# The Effects of Secondary Growth of *Spartina alterniflora* after Treatment on Sediment Microorganisms in the Yellow River Delta

**DOI:** 10.3390/microorganisms10091722

**Published:** 2022-08-26

**Authors:** Shuai Shang, Liangyu Li, Zaiwang Zhang, Yu Zang, Jun Chen, Jun Wang, Tao Wu, Jiangbao Xia, Xuexi Tang

**Affiliations:** 1 School of Biological & Environmental Engineering, Binzhou University, Binzhou 256600, China; 2 College of Marine Life Sciences, Ocean University of China, Qingdao 266005, China; 3 Department of Natural Resources, First Institute of Oceanography, Qingdao 266100, China; 4 Shandong Provincial Key Laboratory of Eco-Environmental Science for Yellow River Delta, Binzhou University, Binzhou 256600, China

**Keywords:** *Spartina alterniflora*, Yellow River Delta, fungal community, bacterial community, intertidal wetlands

## Abstract

As a typical invasive species, *Spartina alterniflora* is widely recognized as one of the primary threats to biodiversity in various habitats, including wetlands. Although the invasion by *S. alterniflora* has been managed in multiple ways, it may reappear after treatment. How *S. alterniflora* affects the soil microbial community in coastal wetlands during its regeneration process has not yet been clarified. Here, rhizosphere soil samples (RSPs) and bulk soil samples (SSPs) were collected in the *S. alterniflora* community and a high-throughput sequencing method was conducted to analyze the composition and diversity of soil microorganisms. Meanwhile, we also obtain the soil physicochemical properties. In the present study, there was no significant difference in the alpha diversity of both bacterial and fungal communities in the SSP and RSP groups. The PCoA (principal coordinate analysis) also showed that the microbial community structure did not differ significantly between the SSP and RSP groups. The results showed that except for pH, the total sulfur (TS) content, total nitrogen (TN) content, and electrical conductivity (EC) did not differ significantly (*p* > 0.05) between the SSP and RSP groups. The composition of the bacterial and fungal community in the rhizosphere of *S. alterniflora* was similar to that found in the surrounding soils. The top two dominant bacterial phyla were Proteobacteria and Desulfobacterota in the present study. Venn diagram results also support this view; most OTUs belong to the common OTUs of the two groups, and the proportion of unique OTUs is relatively small. The LEfSe (LDA effect size) analysis showed that Campylobacterota (at the phylum level) and *Sulfurimonas* (at the genus level) significantly increased in the RSP group, implying that the increased *Sulfurimonas* might play an essential role in the invasion by *S. alterniflora* during the under-water period. Overall, these results suggest that the bacterial and fungal communities were not significantly affected by the *S. alterniflora* invasion due to the short invasion time.

## 1. Introduction

Biological invasion is an essential factor driving global environmental change and has profoundly influenced the biological structure of ecosystems and attracted great attention. The intertidal wetlands are the transition zone between marine and terrestrial environments and are one of the most environmentally stressed regions on Earth. These wetlands are vulnerable to biological invasion due to the alternation of land and water environments [1]. Biological invasion can affect the biodiversity, the ecosystem balances, and the natural wetland landscape succession [2]. As a typical invasive plant species, *Spartina alterniflora* is widely recognized as one of the primary threats to biodiversity in various habitats, including wetlands [3,4]. The invasion by *S. alterniflora* has caused the loss of habitats of native communities and decreased the native species richness, influencing the biodiversity of the intertidal wetland ecosystems [5].

*S. alterniflora*, native to North America, is a perennial and deep-rooted salt marsh grass and is among the most successful invasive species [6]. It was first introduced to China in 1979 for coastal erosion control and sediment stabilization [7]. Due to its greater biomass, higher growth rate, and more vital reproductive capacity, *S. alterniflora* has spread rapidly, out-competing native plants and becoming one of the dominant species in the invaded ecosystems [8,9]. The invasion by *S. alterniflora* can change the circulation process of soil elements in the invasion area. Previous studies found that the sediment organic carbon and total nitrogen (TN) were significantly increased by the invasion of *S. alterniflora* due to the plant deposits [10], leading to a change in the composition of microorganisms in the sediment. It has been reported that *S. alterniflora* provided more nutrition and energy sources, promoted the rhizosphere soil’s microbial community structure, and improved the microbial community’s function, thus creating a better soil condition and facilitating the invasion [11,12]. With the deepening of research, people pay more and more attention to the role of soil microorganisms in the ecosystem. The role of soil microorganisms in the process of biological invasion has also become a research hotspot. It has been reported that *S. alterniflora* provided more nutrition and energy sources, promoted the microbial community structure of the rhizosphere soil, and improved the function of the microbial community, thus creating a better soil condition, which in turn facilitates the invasion [13,14]. Therefore, the invasion by *S. alterniflora* needs to be controlled and studied at the macrolevel and analyzed and summarized at the microlevel, especially with regard to the role and function of microorganisms in the invasion process. By regulating the composition of soil microorganisms, the competitiveness under the ground can change and then affect the survival of the native plants [15]. At the same time, the invasion by *S. alterniflora* also mediates population competition and changes the composition of the benthos [16,17]. The invasion by *S. alterniflora* has been managed in various ways. Many methods have been adopted to control *S. alterniflora* in the YRD, including the cutting plus tilling treatment, the mechanical rolling treatment, and the drowned treatment [18]. Our previous study found that different methods for controlling *S. alterniflora* significantly changed the soil microbial community structure [18].

The Yellow River Delta (YRD) is the youngest wetland ecosystem in China’s warm-temperate zone and it provides multiple ecological services, including soil conservation, water purification, and biodiversity maintenance [19]. Over the past few decades, this delta has experienced significant environmental change due to the invasion by *S. alterniflora*. Many methods have been adopted to control *S. alterniflora* in the YRD. Through monitoring, we found that *S. alterniflora* had a certain proportion of regeneration after treatment. With the progress of microbiome research technology, the study of microorganisms has changed from quantity to quality and from community structure to function. Second-generation sequencing technology and mass spectrum technology derived from acer genome, transcriptome, and the macrometabolism and the macroprotein group can provide a more comprehensive perspective and systematic analysis of the structure and function of microbial groups; thus, the progress of microbial proteomics research methods for the exploration of plant invasion mechanism opens a new route. To explore the influence of *S. alterniflora* regeneration on the YRD, we used a high-throughput sequencing method to analyze the composition and diversity of soil microorganisms. The main objective of this study was to explore how *S. alterniflora* affected soil microorganisms in coastal wetlands during this regeneration process and find the differences in soil properties and microbial communities between the rhizosphere and the bulk soil instead of characterizing them.

## 2. Materials and Methods

### 2.1. Study Area and Soil Sampling

The study was conducted in the Yellow River Delta (37°16′ N–38°16′ N, 118°20′ E–119°20′ E) in Shandong Province, China. This area has a typical continental monsoon climate, characterized by four distinct seasons. The mean annual temperature and precipitation are 12.3 °C and 542.3 mm, respectively. Salty soil is the main soil type in this area. The vegetation that is widely distributed includes *Suaeda salsa* and *Tamarix chinensis* [20].

In this area, *S. alterniflora* is discontinuously distributed in the invasive range. The density of *S. alterniflora* is 80–110 per square meter in the study area. The *S. alterniflora* was controlled by the cutting plus tilling treatment in August 2021 and the newly invasive *S. alterniflora* first appeared in April 2022. In May 2022, four sampling sites were randomly selected and there were two plots set at each site. Each plot was 1 m × 1 m and the distance between the adjacent plots in the same site was 100 m. Two rhizosphere and bulk soil samples were collected from each plot, respectively, and a total of 16 soil samples were obtained. After removing the soil that was indirectly attached to the root by gently shaking the plant, the rhizosphere soil was collected by brushing the soil from the root surface with a sterile soft-bristled paint brush, and the bulk soil at the 0–10 cm layer was collected using the soil auger. The rhizosphere soil samples were marked as the RSP group and the bulk soil samples were marked as the SSP group. The SSP samples were collected from the bulk soil and the plot was carefully checked to see whether there were roots/rhizomes in the bare soil area. The sampling site was abandoned immediately if any roots/rhizomes were found. The RSP samples were taken according to the rhizosphere microbial sampling method [15]. After collection, all samples were placed in a dry ice box and brought back to the laboratory. All soil samples were divided into two parts. One part was air dried and stored at 4 °C for determining soil physicochemical properties, and the other was stored at −80 °C for DNA extraction.

### 2.2. Soil Physicochemical Properties Analysis

Soil pH was measured in the supernatant with 1:5 soil–water mixtures by a pH meter (Sartorius PB-10, Gottingen, Germany). The total nitrogen (TN) was measured on an Elemental Analyzer (CHOS, Elemental Analyzer, Vario EL, Germany). Electrical conductivity (EC) was measured using a conductivity meter with dry soil–water ratio of 1:5 (Hanna HI98192, Italy). The sulfur was determined by an inductively coupled plasma atomic emission spectrometry (ICP/AES).

### 2.3. Soil DNA Extraction, Amplification, and Sequencing 

Total genomic DNA was extracted from soil samples using TGuide S96 Magnetic Soil DNA Kit (DP812, Tiangen Biotech Co., Ltd., Beijing, China) according to the manufacturers’ instructions. Then, the extracted DNA quality and purity were measured by agarose gel electrophoresis and an ultraviolet spectrophotometer, respectively. The V3-V4 region of the 16S rRNA gene was amplified using forward primers 338 (5′-ACTCCTACGGGAGGCAGCA-3′) and reverse primers 806 (5′-GGACTACHVGGGTWTCTAAT-3′). The ITS1 region of the fungal rRNA gene was amplified using forward primers ITS1 (5′-CTTGGTCATTTAGAGGAAGTAA-3′) and reverse primers ITS2 (5′-GCTGCGTTCTTCATCGATGC-3′). Polymerase chain reaction (PCR) was performed under the following conditions: the initial denaturation was at 95 °C for 5 min, followed by 25 cycles at 95 °C for 30 s, 50 °C for the 30 s, 72 °C for 40 s, and the final extension was at 72 °C for 7 min. The PCR reaction volume was 20 µL: 13.25 µL H_2_O, 2.0 µL 10 × PCR ExTaq Buffer, 0.5 µL DNA template, 1.0 µL prime1, 1.0 µL prime2, 2.0 µL dNTP, and 0.25 µL ExTaq. PCR-amplified products were detected insusing 1.8% agarose gel electrophoresis, purified using VAHTSTM DNA Clean Beads (Vazyme, Nanjing, China), and then quantified using a Quant-iT™ dsDNA HS Assay Kit (Thermo Fisher Scientific, Waltham, MA, USA). Finally, paired-end sequencing was conducted on an Illumina Novaseq 6000 platform at Biomarker Technologies Corporations (Beijing, China). The raw sequence data in the present study were deposited at the Sequence Read Archive (SRA) database of NCBI under accession numbers SAMN29837827 to SAMN29837842 for bacteria and SAMN29837843 to SAMN29837858 for fungi.

### 2.4. Sequence Processing and Statistical Analysis

To obtain high-quality clean reads, the raw reads were filtered using Trimmomatic v0.33, and primer sequences were identified and cut using cutadapt 1.9.1 [21,22]. Then, the clean reads were merged depending on overlap using Usearch v10 [23]. Finally, the denoising and removal of chimeric sequences were conducted to obtain non-chimeric reads using DADA2 in QIIME2 2020.6 [24,25]. The non-chimeric reads were clustered into the operational taxonomic unit (OTU) at the 97% similarity level, whereafter the final OTUs that were used were purified at the 0.005% threshold. Bacterial and fungal taxonomic identities were determined using the SILVA v138 database [26]. Alpha diversity indices (AEC, Chao1, Shannon and Simpson indices) and beta diversity were calculated using QIIME2 software (version 2020.6) [25]. Venn diagrams were constructed to show the shared and unique OTUs between the SSP and RSP groups. The principal coordinate analysis (PCoA) based on the Bray–Curtis distance was used to visualize the difference in the microbial community structure between the two groups. LDA effect size (LEfSe) was used to identify the significant difference in the bacterial and fungal community composition at the different taxonomic levels. The criterion for LEfSe was set as *p <* 0.05, LDA > 4.0. Redundancy analyses (RDA) were conducted to investigate the relationship between the soil physicochemical properties and the microbial community composition. All figures were constructed using R v3.4.4 [27]. The differences in the soil physicochemical properties and the soil alpha diversity indices were tested by Student’s *t*-test using SPSS 23.0 (IBM SPSS Inc., Chicago, IL, USA). The difference in the microbial community structure was tested using permutational multivariate analysis of variance (PERMANOVA). Significant differences were defined as *p <* 0.05. 

## 3. Results

### 3.1. Soil Physicochemical Properties

The soil physicochemical properties of samples were analyzed in the present study. Our results showed that the average values of all soil physicochemical properties were higher in the SSP group than in the RSP group. Soil pH changed significantly (*p* < 0.05), whereas the sulfur content, the total nitrogen (TN) content, and the electrical conductivity (EC) did not differ significantly (*p* > 0.05) between the SSP and RSP groups (Table 1).

### 3.2. Bacterial and Fungal Community Diversity 

For the bacterial community, 1,273,100 raw reads were obtained, and after filtering, 1,264,325 clean reads were obtained. For the fungal diversity, 1,280,048 raw reads were obtained and after filtering, 1,266,239 clean reads were obtained. Diversity indices (Shannon and Simpson) and richness indices (Chao1 and ACE indices) were calculated to analyze microbial alpha diversity. In the present study, the bacterial and fungal community diversity did not differ significantly (*p* > 0.05) between the SSP and RSP groups (Table 2 and Table 3). The Chao1, Shannon, and Simpson indices for the bacterial community were higher in the SSP group, whereas the ACE index was higher in the RSP group. All diversity and richness indices for the fungal community were higher in the SSP group

**Figure 1:**
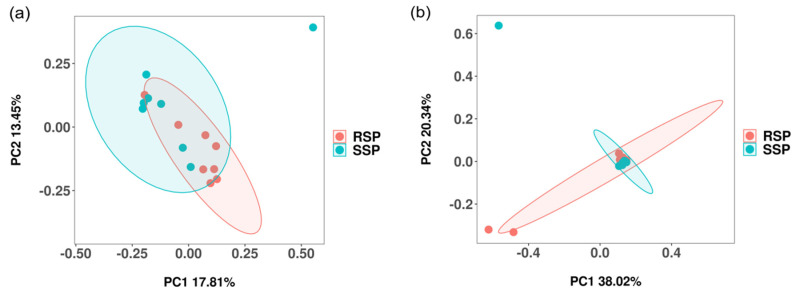
The PcoA based on the Bray–Curtis distance showing the variation in the bacterial (**a**) and the fungal (**b**) community structure. Different colors represent the samples from different groups.

The principal coordinate analysis (PCoA) based on the Bray–Curtis distance was used to exhibit the differences in bacterial and fungal community structure between the SSP and RSP groups. For the bacterial community, the first principal component (PC1) explained 17.81% of the variation and the second principal component (PC2) explained 13.45% of the variation, respectively (Figure 1a). PC1 had the greatest impact. In total, 31.26% of the variation was explained by the two principal components. For the fungal community, PC1 explained 38.02% of the variation and PC2 explained 20.34% of the variation (Figure 1b). PC1 had the greatest impact. In total, the two principal components explained 58.36% of the variation. To statistically support the clustering of bacterial and fungal communities between the two groups, the *p*-value was calculated via PERMANOVA. The community structure of bacteria and fungi did not exhibit a significant difference between the SSP and RSP groups (R^2^ = 0.080, *p* > 0.05; R^2^ = 0.081, *p* > 0.05).

**Figure 2:**
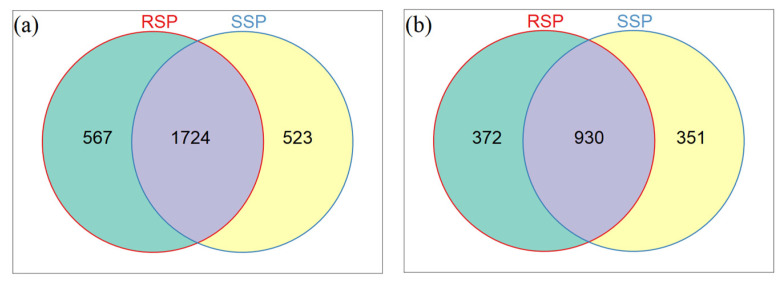
Venn diagrams showing the number of shared and unique OTUs between groups. (**a**) Bacteria and (**b**) fungi. Each circle represents sampled compartments. Values within intersections represent shared OTUs, values outside intersections represent unique OTUs.

The Venn diagram showed the number of shared and unique OTUs between the SSP and RSP groups. For the bacterial community, there were 1724 shared OTUs, and 567 unique OTUs in the RSP group and 523 unique OTUs in the SSP group, respectively (Figure 2a). For the fungal community, there were 930 shared OTUs, 372 unique OTUs in the RSP group and 351 unique OTUs in the SSP group, respectively (Figure 2b).

### 3.3. Bacterial and Fungal Community Composition

**Figure 3:**
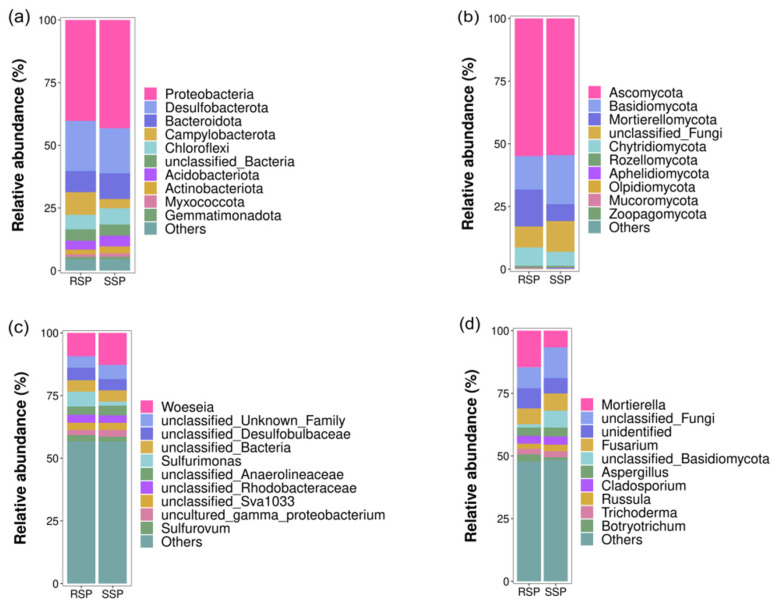
The relative abundance of the top 10 bacterial (**a**,**c**) and fungal (**b**,**d**) taxa at the phylum (**a**,**b**) and genus (**c**,**d**) level.

The relative abundance of different bacterial and fungal taxa at the phylum and genus level was shown in Figure 3. The dominant bacterial phyla (relative abundance of more than 1%) were Proteobacteria (40.29–43.20%), Desulfobacterota (17.97–19.95%), Campylobacterota (3.69–9.04%), Bacteroidota (8.44–10.25%), Chloroflexi (5.83–6.50%), Acidobacteriota (3.50–4.23%), Actinobacteriota (1.88–2.81%), and Myxococcota (1.03–1.36%) (Figure 3a). The relative abundance of Proteobacteria was the highest, followed by the relative abundance of Desulfobacterota, in both the SSP and RSP groups. At the genus level, except for the unclassified genera, the dominant bacterial taxa were *Woeseia* (9.34–12.75%), *Sulfurovum* (1.94–2.53%), and *Sulfurimonas* (1.65–5.92%) (Figure 3c). *Woeseia* was the most abundant genus in both the RSP and SSP groups. For the fungal community, the dominant phyla were Ascomycota (54.52–54.94%), Basidiomycota (13.30–19.59%), Mortierellomycota (6.73–14.73%), and Chytridiomycota (5.60–7.29%) (Figure 3b). Ascomycota was the most abundant phylum in both the RSP and SSP groups. Except for the unclassified genera, the dominant fungal genera were *Fusarium* (6.32–6.87%), *Mortierella* (6.69–14.61%), *Aspergillus* (3.32–3.62%), *Cladosporium* (3.09–3.22%), *Russula* (2.05–2.63%), *Trichoderma* (2.22–2.38%), and *Botryotrichum* (1.08–2.84%) (Figure 3d). *Fusarium* was the most abundant genus in the SSP group, while *Mortierella* had the highest relative abundance in the RSP group.

### 3.4. LefSe Analysis

**Figure 4:**
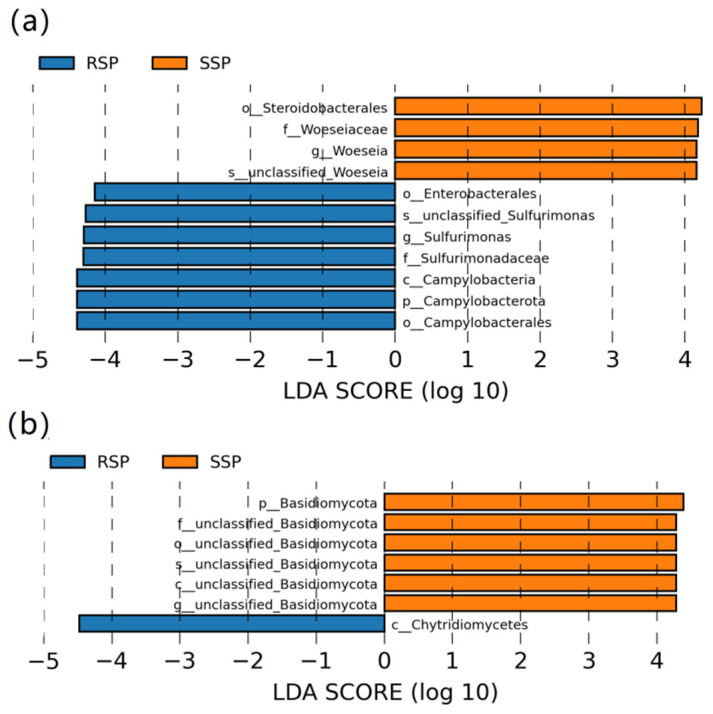
LEfSe analysis showing the significant difference at different bacterial (**a**) and fungal (**b**) taxonomic levels between the SSP and RSP groups. Different colors represent different groups.

The LefSe analysis was used to compare the significant difference in the relative abundance of some taxa between the two groups (LDA > 4.0, *p <* 0.05). For the bacterial community, the relative abundance of the phylum Campylobacterota, the class Campylobacteria, the order Enterobacterales and Campylobacterota, the family Sulfurimonadaceae, and the genus *Sulfurimonas* were significantly higher in the RSP group than in the SSP group. The relative abundance of the order Steroidoloacterales, the family Woeseiaceae, and the genus *Woeseia* significantly increased in the SSP group (Figure 4a). Except for the unclassified taxa for the fungal community, only the phylum Basidiomycota rose markedly in the SSP group and the class Chytridiomycetes significantly increased in the RSP group (Figure 4b).

### 3.5. Redundancy Analysis (RDA) Analysis

**Figure 5:**
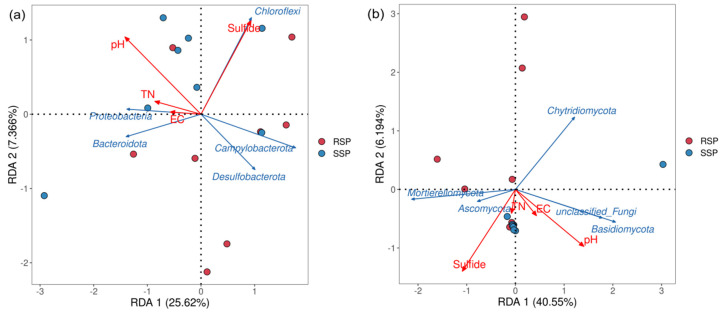
RDA of the relationship between the soil physicochemical properties and bacterial (**a**) and fungal (**b**) genera. RSP, rhizosphere soil samples; SSP, bulk soil samples; TS, total sulfur; TN, total nitrogen; EC, soil electrical conductivity.

RDA was performed to explore the relationship between soil physicochemical factors and microbial community structure. The top five bacterial and fungal phyla in relative abundance and the soil physicochemical factors of sulfur, pH, TN, and EC were used as parameters, respectively. Proteobacteria was strongly correlated with the TN and EC for the bacterial community and Chloroflexi was strong in sulfur content. The two axes of the RDA explained 25.62% and 7.366% of the bacterial community variation, respectively (Figure 5a). For the fungal community, Basidiomycota was strongly correlated with pH. The two axes of the RDA explained 40.55% and 6.194% of the fungal community variation, respectively (Figure 5b)

## 4. Discussion

Invasive plants can affect the structure and function of the microbial community of invaded areas in various ways. Invasive plants can release chemical residues and change the soil matrix and physical and chemical properties [28]. Invasive plants break the original material circulation pattern and affect soil microorganisms’ structure composition and function. They also change the processing, decomposition, and transformation of rhizosphere microorganisms of native plants, thus affecting the absorption of nutrients, energy flow, and genetic information transfer of native plants [29]. Researchers found that the plant microbiome mainly drives short-term changes in the plant environment. In contrast, long-term changes are driven by ecological and evolutionary interactions between the plant microbiome and its host [30]. Thus, exploring the dynamic changes in soil microbial community structure of intertidal wetlands under the *S. alterniflora* invasion is meaningful. *S. alterniflora* has a high tolerance and adaptability to environmental stressors.

In this study, the alpha diversity indices of the bacterial and fungal communities did not differ significantly between the SSP and RSP groups. Although diversity did not differ significantly between the SSP and RSP groups, higher values in the RSP group suggested that the microbial communities were influenced by plant root exudates and soil physicochemical properties. The rhizosphere microorganisms mainly rely on plant root exudates as a food source, and this is closely related to the diversity of microbial communities. Conversely, microorganisms in the bulk soil are lightly affected by plant root exudates, with a consequent lower level of microbial diversity [31]. Our result was not consistent with the above finding. It was reported that the decreased pH could inhibit the bacterial enzymatic and metabolic activities, which is not conducive to the growth of bacteria [32]. The pH value in the RSP group decreased significantly compared to the SSP group, which may explain the lower diversity of the rhizosphere bacterial community. A previous study found that a higher microbial alpha diversity might improve the resilience of *S. alterniflora* and be an effective strategy for successful invasion [33]. We speculated that the bacterial and fungal communities were not significantly affected by the *S. alterniflora* invasion due to the short invasion time after the control treatment in August 2021. Venn diagram results also support this view, showing that most OTUs belong to the common OTUs of the two groups and the proportion of unique OTUs is relatively small.

The relationship between the soil’s physical and chemical properties and the plants and microorganisms was complex. Our results showed that the sediment pH was significantly different between the groups. The sediment pH was changed by the *S. alterniflora* invasion with values decreasing, similar to the previous study [34,35]. We speculated that *S. alterniflora* was accompanied by the release of H^+^ in sediments, and that caused a decrease in pH. The sulfur content, TN content, and EC did not differ significantly (*p* > 0.05) between the SSP and RSP groups. A previous study found that *S. alterniflora* invasion could substantially affect the sulfur [15] and TN content [35]. At the same time, the soil’s physical and chemical properties varied considerably within the group. We speculated that the soil’s physical and chemical properties were not significantly affected due to the short invasion time of the revived *S. alterniflora*. 

For the bacteria, the dominant phyla in wetland soil were Proteobacteria, Bacteroidota, Chloroflexi, Acidobacteriota, and Actinobacteriota, and the frequently observed dominant genera were *Woeseia*, *Sulfurovum,* and *Sulfurimonas*. For fungi, the dominant phyla were Ascomycota, Basidiomycota, and Chytridiomycota [18]. The above microbial taxa were also found in both the SSP and RSP groups. The top two dominant bacterial phyla were Proteobacteria and Desulfobacterota in the present study, whereas Proteobacteria and Bacteroidota were detected as the most abundant bacterial phyla in our previous study [15]. Though the composition of the bacterial community in the rhizosphere of *S. alterniflora* was similar to that in the surrounding sediments, some differences were still identified in composition. The LEfSe analysis showed that the Campylobacterota (at the phylum level) and the *Sulfurimonas* (at the genus level) significantly increased in the RSP group. Previous studies found that *Sulfurimonas* reduced sulfur compounds as electron donors and nitrate, nitrite, and oxygen as electron acceptors, and *Sulfurimonas* used CO_2_ as the sole carbon source [36]. Zhang et al. found that *Sulfurimonas* has a better adaptation to lower oxygen concentrations and lower available carbon conditions [37]. *S. alterniflora* is distributed in an intertidal zone that was submerged in seawater for quite a long time. Thus, we speculated that the increased *Sulfurimonas* might play an essential role in the invasion by *S. alterniflora* during the under-water time. Physical and chemical properties significantly affect the structure of the microbial community. In the present study, the RDA results showed that the fungal phylum Basidiomycota was strongly correlated with pH, which was consistent with previous studies, demonstrating that the richness of Basidiomycota was explained by a positive response to soil pH [38,39]. 

## 5. Conclusions

In the present study, we explored the effects of the secondary growth of *S. alterniflora* on sediment microorganisms in the Yellow River Delta to deeply understand the scientific basis for the rational management of *S. alterniflora*. In conclusion, other than the pH, the sulfur content, TN content, and EC did not differ significantly between the SSP and RSP groups. The top two dominant bacterial phyla were Proteobacteria and Desulfobacterota in the present study. The Venn diagram results also support this view, showing that most OTUs belong to the common OTUs of the two groups, and the proportion of unique OTUs is relatively small. Additionally, the bacterial and fungal communities were not significantly affected by the *S. alterniflora* invasion due to the short invasion time. 

## Figures and Tables

**Table 1:** Changes in soil physicochemical properties between the SSP and RSP groups.

Groups	Sulfur (mg/kg)	TN (mg/kg)	pH	EC (ms/cm)
SSP	1.39 ± 0.11 a	232.88 ± 40.20 a	8.26 ± 0.49 a	2.49 ± 0.21 a
RSP	1.33 ± 0.15 a	169.13 ± 29.38 a	7.89 ± 0.49 b	1.98 ± 0.15 a

TN means total nitrogen and EC means electrical conductivity. Values are the means ± standard error (*n* = 8). Lowercase letters in the same column indicate significant differences between the two groups (*p <* 0.05).

**Table 2:** Bacterial diversity indices in different groups.

Groups	ACE	Chao1	Simpson	Shannon
SSP	707.73 ± 35.83	712.83 ± 36.53	0.9965 ± 0.0004	8.79 ± 0.11
RSP	708.90 ± 20.64	709.83 ± 20.79	0.9955 ± 0.0005	8.68 ± 0.08

Values are the means ± standard error (*n* = 8).

**Table 3:** Fungal diversity indices in different groups.

Groups	ACE	Chao1	Simpson	Shannon
SSP	421.61 ± 45.59	421.69 ± 45.61	0.9809 ± 0.0057	7.24 ± 0.28
RSP	409.13 ± 28.15	409.84 ± 28.19	0.9586 ± 0.0121	6.81 ± 0.27

Values are the means ± standard error (*n* = 8).

## Data Availability

The data that support the findings of this study are available in NCBI (https://www.ncbi.nlm.nih.gov/ (accession number (SAMN29837827-SAMN29837858) (accessed on 20 July 2022).
